# Management of Dental Avulsion Injuries: A Survey of Dental Support Staff in Cairns, Australia

**DOI:** 10.3390/dj9010004

**Published:** 2020-12-30

**Authors:** Yannis Abraham, Roshini Christy, Americo Gomez-Kunicki, Ting Cheng, Silvia Eskarous, Verona Samaan, Ahsen Khan, Amar Sholapurkar

**Affiliations:** 1 College of Medicine and Dentistry, James Cook University, Cairns, QLD 4870, Australia; Roshini.Christy@my.jcu.edu.au (R.C.); Americo.GomezKunicki@my.jcu.edu.au (A.G.-K.); Ting.Cheng@my.jcu.edu.au (T.C.); Silvia.Eskarous@my.jcu.edu.au (S.E.); Verona.Samaan@my.jcu.edu.au (V.S.); 2 Private Practice, Corrimal, NSW 2518, Australia; Ahsen.Khan@my.jcu.edu.au

**Keywords:** avulsion, dental trauma, emergency management, pediatric dentistry, public health dentistry

## Abstract

Background/Aim: The aim of this study was to assess the knowledge of dental support staff in providing appropriate first-aid advice regarding dental avulsion emergencies. Methods: This study was reported according to the Strengthening the Reporting of Observational Studies in Epidemiology (STROBE) guidelines for cross-sectional studies. Dental support staff (includes dental assistants, administrative staff and other non-clinical staff) were contacted and data were collected from 50 private dental clinics across the Greater Cairns Area, Queensland, Australia. These data were collected through an online survey throughout 2020. Descriptive statistics and Pearson’s Chi-squared test was used to analyze the data and any associations between categorical outcomes. Results: This survey yielded a response rate of 34.1% with a margin of error of 10.3%. More than four-tenths of participants (42%) reported that they had received some form of dental avulsion management training previously. All but five participants (92%) denoted that they would immediately replant an avulsed permanent tooth. More than half of all participants would choose to rinse a soiled avulsed tooth with fresh milk (55%) and transport that tooth in fresh milk (65%) should they not be able to replant the tooth at the site. Almost nine in every ten participants (85%) expressed willingness to further their training in this area. Knowledge in replanting avulsed permanent teeth was found to be significantly impacted by gender, age, years of experience and participation in formal avulsion training. Male participants were found to be significantly more likely (*p* = 0.025) to replant a permanent avulsed tooth than their female counterparts. Participants who were 40 years of age and above were found to be significantly more likely to choose fresh milk to transport avulsed teeth (*p* = 0.0478). Older participants (*p* = 0.0021), alongside those who had greater years of experience (*p* = 0.0112) and those who had undertaken formal avulsion training (*p* = 0.0106) were all significantly more likely to express greater confidence in their ability to manage dental avulsion injuries. Participants who had previously received some form of education regarding avulsion injury management were also most likely to warrant further education and training in this area (*p* < 0.0001). Conclusion: This study demonstrated that dental support staff in the Greater Cairns Area seem to have a fair grasp of first-aid knowledge regarding the management of dental avulsion injuries. This result indicates that this knowledge has been picked up through years of experience, rather than a formal education. Despite this, one would expect people who work in the dental industry to be able to provide accurate and appropriate assistance during dental emergencies, hence, further training is warranted to ensure optimum patient outcomes.

## 1. Introduction

Avulsion is a critical dental injury in which a tooth is completely removed from its socket due to trauma, making up 0.5–3% of all dental injuries [1]. In an avulsion injury, damage occurs to the periodontal ligament, the neurovascular bundle, cementum, alveolar bone and gingiva [1,2]. Complex treatment is therefore necessary as multiple components of the tooth are affected. Moreover, instant action taken at the site of incident will improve overall tooth prognosis [1]. If immediate action is not taken, long-term consequences, including post-operative treatment complications and inflammatory or replacement root resorption, may occur [2]. Injuries sustained to permanent anterior teeth occur frequently during childhood between the ages of 7 and 10, in which facial growth and psychosocial development is of utmost importance [2]. The teeth most commonly affected are maxillary central incisors, followed by maxillary lateral incisors and then mandibular central incisors [1,3]. Avulsion injuries are often a result of falls, sometimes during sporting activities or traffic accidents. Malocclusions such as increased overjet, protrusion and poor lip coverage of the maxillary anterior dentition can also predispose one to higher risk of dental trauma injuries [4].

According to the International Association of Dental Traumatology (IADT), avulsion management in the clinic should strive to prevent infection of the pulpal tissues [5]. Teeth that reside extra-orally for longer than 60 min usually have a poor long-term prognosis [6]. Thus, all permanent avulsed teeth should be re-implanted almost instantaneously [5,6]. If immediate reimplantation of the tooth is not achievable, the avulsed tooth should be stored in milk, a saline or sodium fluoride so as to preserve the biological components of the tooth [5,6,7]. It was noted that refrigerated milk is a suitable medium, due to its ideal pH (6.5–7.2), temperature (<25 °C) and availability [7]. A study by Khinda et al. discussed other liquid mediums including, Hank’s balanced salt solution (HBSS), tooth rescue box (Dentosafe) and coconut water. They found that all these liquids were superior storage medium for avulsed teeth than dairy milk [6]. However, these products are not as practical due to lack of availability at the time of tooth avulsion injury [6]. Storage mediums such as tap water, saliva, alcohol and saline solution have non-ideal pH, thus are not recommended [6].

Furthermore, the vessel that encloses the storage medium is an additional factor which contributes to the preservation of the avulsed tooth [8]. This vessel should possess the following characteristics: sterile, resistant to cracks/fractures, provide a tight seal and made of biocompatible material [9]. Additionally, all avulsed teeth should be handled with care and held only from the crown of the tooth [7,8,9]. If the avulsed tooth is held from the root tip, then damage to the periodontal ligament cells may ensue [7,8,9].

According to a study conducted by Cvek et al., [7] the prognosis of an avulsed tooth is largely determined by the preservation of the periodontal ligament fibers as this component serves as the connection between the avulsed tooth and the bony socket [7]. Therefore, the preservation of the periodontal ligament cells residing on the root surface of the tooth is of utmost importance [10]. They begin to disintegrate as early as 5 min after an avulsion injury [11]. If an avulsed tooth is not replanted before 60 min, the viability of the periodontal ligament cells greatly diminishes, and the chance of survival reduces [9,10,11]. This will then produce an avenue for unwarranted complications such as root resorption [12]. Despite this, it should be noted that most dental practitioners experience considerable difficulty replanting avulsed teeth upon immediate clinical presentation [13]. Additionally, replantation without sufficient anesthesia has shown to be distressing for the affected patient, particularly if patient cooperation is unachievable [11,12,13].

According to the American Dental Association, to ensure the survival of an avulsed tooth, avulsion management in the clinic should be segregated into three main categories [7]:

1. Reimplantation of the avulsed tooth promptly.
2. A subsequent appointment involving a comprehensive clinical and radiographic assessment of the tooth in question.
3. Root canal therapy if indicated.

Furthermore, the way in which the avulsed tooth is re-implanted is highly important, as incorrect placement can increase the risk of root resorption [14]. The type of splint used must be carefully considered as an inflexible splint can warrant unfavorable outcomes such as ankylosis [15]. It should be noted that with all avulsion injuries the risk of aspiration of the avulsed tooth is probable and thus it is pertinent that general dentists refer the patient for chest and bowel X-rays, as a precautionary measure [16].

An investigation was conducted in Pub-Med, Ovid databases and through hand search to explore existing literatures; the key words ‘dental staff’, ‘avulsion injury’ and ‘avulsion management’ were utilized. Only one study about dental avulsion injuries was conducted in Australia; however, this study targeted primary school teachers [17]. Furthermore, it was clear that there has been very minimal research conducted globally in evaluating the knowledge of dental staff with regards to avulsion injuries. One study carried out by Halawany et al. [18] in Saudi Arabia assessed the knowledge of dental assistants with regards to avulsion management. A closer inspection of the study highlighted the heightened awareness of dental practitioners in comparison to the dental assistants [18]. Additionally, another study conducted in Southern California by Cohenca et al. [19] assessed the knowledge of oral health professionals, mostly dental practitioners, on the guidelines for management of avulsed teeth. The authors revealed varying degrees of knowledge amongst participants on this topic [19].

Research into dental avulsion is a topic of significance, as it was found that approximately 5% of injuries sustained to the human body arise from the oral cavity [20]. Additionally, studies have noted that dental trauma management within a hospital setting tends to be very expensive and time consuming [20]. According to a survey conducted by Yigit et al. [21], dental trauma injuries are responsible for approximately 7% of the injuries encountered in the emergency department. Ultimately, our research efforts endeavored to assess the knowledge of dental support staff (DSS) in providing appropriate advice regarding dental avulsion emergencies. Dental support staff is defined as dental assistants, oral health nurses, dental administrators and clinical directors. Importantly, DSS are often the first responders to the avulsion emergency, before the dentist and specialist. Thus, knowledge and competency of the DSS has a great impact on overall management of avulsion injuries. This research is distinctive in that no paper in Australia has specifically assessed the knowledge DSS have on management of avulsion injuries.

This research aims to assess the knowledge and understanding of Australian DSS in the Greater Cairns Region with regards to tooth avulsion management. It is hypothesized that Australian DSS’ understanding of dealing with avulsion injuries will be insufficient. Seeing that this would be the first study conducted on this topic in Australia, the results obtained would assist in devising strategies that aim to increase DSS’ awareness and competency in managing such dental emergencies.

## 2. Methodology

Approval of this study was gained from the Human Research Ethics Committee of James Cook University (H8112). This study is reported according to the Strengthening the Reporting of Observational Studies in Epidemiology (STROBE) guidelines for the presentation of cross-sectional studies [22]. It comprised of a questionnaire that analyzed the knowledge of dental avulsion amongst DSS. The target population for this study was limited to DSS currently employed by dental clinics in the Greater Cairns Region, Far North Queensland, Australia.

Exclusion criteria included registered Australian general dentists, registered dental specialists and unemployed DSS. An online survey was created on Survey Monkey to email clinics and preserve social distancing protocols (COVID-19).

Data collection occurred over a period of one month starting from the 14 July 2020. A Yellow Pages search for dental clinics in the Greater Cairns region yielded 88 search results. These clinics were contacted via telephone and presented with a brief summary of the study in order to gain consent to their participation in the study.

Of the 88 clinics contacted, 50 clinics agreed to receive the online questionnaire. Furthermore, each clinic contacted was questioned on the number of DSS that were currently employed. Responses ranged from 2 to 4 DSS/clinic with an average of 2 DSS/clinic.

A detailed online three-part survey was developed for use in this study via SurveyMonkey. The online questionnaire was compared and modified from the questionnaire utilized by Cohenca et al. [23] for their study of specialized oral health professionals, general dentists and dental assistants. The questionnaire was further marked against the guidelines set by the IADT to validate its format [24]. It was further scrutinized by the designated research project coordinator and sent to a statistical specialist and clinical dentists in order to be validated. In addition to this, a pilot study was proposed, which consisted of at least five responses from DSS to assess the effectiveness of the online survey [25,26].

Data collection was de-identified thus maintaining the anonymity of the participants and preventing any form of bias. Questions adhered to the following themes:

1. Demographics including gender, age, years of experience and level of qualification.
2. Multiple choice and open-ended questions in relation to the knowledge and management of avulsed teeth.
3. Dental avulsion education, including questions regarding prior training or education about dental avulsion.

All questions were close ended with multiple choice options given for participants to choose from. (Refer to Appendix A)

## 3. Statistical Method

Both the Australian Health Practitioner Regulation Agency (AHPRA) and the Australian Dental Association (ADA) were contacted via email and telephone to enquire whether an official registry of DSS existed and was available to calculate sample size. However, we were informed that such a registry was not available for access. Due to this, the sample size was calculated relating to the average number of 2 DSS employed per clinic contacted in the Greater Cairns Region.

Based on the 88 dental clinics identified via Yellow Pages and contacted by telephone, a total of 176 DSS was estimated and a required sample size of 63 respondents was calculated. This accounted for a 95% confidence level, estimated proportion of 50% and a standard error of 10% [27].

However, upon contacting all available clinics, 38 of the 88 clinics either refused or failed to respond, leading to only 50 clinics consenting to receive the online survey. Within this sample group, a total of 60 responses were obtained. A total respondent rate of 34.1% was obtained, indicating a 10.3% margin of error throughout the results with a 95% confidence interval [28].

Descriptive statistics were used for analysis of raw data collected from the survey. Univariate associations between a categorical outcome and the variables under consideration were evaluated using Pearson’s Chi-squared test. A cut-off of *p* ≤ 0.05 was considered to be statistically significant, with a 95% confidence interval. GraphPad Prism Version 8.0.0 for Mac (San Diego, CA, USA) was used to analyze the data.

## 4. Results

Of the estimated 176 DSS from 88 dental clinics contacted, 60 responses were collected via online survey over a duration of one month. Of the 60 respondents, 88.3% were female and 11.67% were male (see Table 1). Most participants were in the 20–39-year age group (55%). The majority of the dental support staff reported to have 5–10 years (30%) or more than 20 years of experience (30%). Certificate III in Dental Assisting (48.33%) was the most common qualification amongst participants. Seven participants (11.67%) had higher qualifications of bachelor’s degree in Oral Health and 6.67% of the respondents had qualifications other than a certificate in dental assisting, overseas dental degree or a bachelor’s degree. Half of all participants worked full time, 18.3% part-time and 35% as casual employees.

Of those surveyed, 25 staff (41.7%) had received previous avulsion training and only 13 (21.7%) had been supplied with an educational brochure about the management of dental avulsion (refer to Table 2).

Amongst respondents, 55 (91.7%) understood the urgency for immediate dental treatment and were aware of the need to replant permanent teeth (Table 3). Further questions were asked to assess the general knowledge about avulsion and its management as shown in Table 3 and Table 4. Most participants (55%) responded appropriately, stating that a soiled avulsed tooth should be rinsed with fresh milk, while 30% participants stated that an avulsed tooth should be cleaned with the patient’s own saliva. The results showed that 53 respondents (88.3%) were aware that it was best to handle an avulsed tooth from the crown.

The percentage distribution of responses about knowledge on subsequent avulsion management is shown in Table 4. More than half of the participants (55%) responded that the use of a liquid medium would be the best transportation technique, while 35% chose to keep the tooth in the patient’s mouth between cheek and teeth. The most selected liquid medium used to transport an avulsed tooth was fresh milk (65%) followed by patient’s saliva (28.3%). Only 10 respondents (16.7%) considered themselves as being very confident in managing dental avulsion, while the majority (66.7%) reported to have some degree of confidence. However, 16.7% were not confident at all (Table 5). Most respondents (86.7%) showed a willingness to receive further avulsion training.

Analysis of correlations between participants’ demographic information and their management of dental avulsion indicated that knowledge to replant an avulsed permanent tooth was related to their gender, age, years of experience in dentistry and exposure to dental avulsion training. Male participants were found to be significantly more likely (*p* = 0.025) to replant a permanent avulsed tooth. Participants who were 40 years of age and above were found to be significantly more likely to choose fresh milk to transport avulsed teeth (*p* = 0.0478). Older participants (*p* = 0.0021), alongside those who had greater years of experience (*p* = 0.0112) and those who had undertaken formal avulsion training (*p* = 0.0106), were all significantly more likely to express greater confidence in their ability to manage dental avulsion injuries. Participants who had previously received some form of education regarding avulsion injury management were also most likely to warrant further education and training in this area (*p* < 0.0001).

## 5. Discussion

Dental avulsion is the most time-dependent emergency in dentistry threatening tooth mortality. If an avulsed permanent tooth is not re-implanted as soon as possible, or transported to care in an appropriate medium, the long-term prognosis is immediately compromised. Despite varying knowledge reported in previous studies [29,30,31,32,33,34,35,36,37], immediate referral to emergency dental treatment is accepted as a universal response. As such, the first responders in this situation are the DSS, followed by dentists and dental specialists [33].

DSS form an integral part of the team which assists in overall provision of dental treatment, especially in emergency situations. Therefore, it is imperative that DSS are educated and up to date on knowledge of dental avulsion and its emergency management protocol. In this study, 83.3% of DSS had attained a qualification of Certificate III in Dental Assisting or higher (Certificate IV in Dental Assisting, Bachelor of Oral Health or Overseas Dental Degree). Despite this high proportion of educated DSS, 58.3% had not received any previous avulsion training and an even higher 78.3% had not seen posters or educational leaflets on avulsion management. Despite this, knowledge of initial management of avulsion was found to be adequate. Thus, a participant’s previous exposure or non-exposure to avulsion training did not significantly affect their initial management. However, participants with a higher level of education, like Bachelor of Oral Health and overseas dental education, had the greatest number of appropriate responses to the questions. Participants with lower qualifications, such as DSS with certificate III and IV of dental assisting and those with non-dental education, provided a greater number of incorrect responses. This is consistent with a finding by Halawany et al. [18], where participants with an overseas dental degree had superior knowledge compared to those without, in management of avulsion injuries. Thus, Halawany et al.’s study on dental assistants displayed the positive effect of a formal dental education on retention of knowledge and management of avulsion [18].

Age (*p* = 0.0478) and years of experience (*p* = 0.0112) were found to have a significant impact on a participant’s choice of liquid medium in which to carry an avulsed tooth. Participants above the age of 40 and those with more years of experience were significantly more likely to choose fresh milk. This suggests that despite a lack of formal training, participants’ life experience and professional experience can have a critical impact in maximizing a successful treatment outcome.

These results differ from Halawany et al.’s study in that a greater percentage of participants had less than 5 years’ experience (41.8%, compared to this study’s 20%), yet a larger percentage of respondents (79.1%) had the appropriate knowledge of tooth avulsion management [21].

Despite the majority of responses to management questions on dental avulsion being accurate, only 10 participants (16.7%) reported feeling very confident in managing the emergency. The remaining 83.3% were only confident to some degree or not confident at all (66.67% and 16.67% respectively). Being the first to attend to incoming phone calls and talk to patients upon presentation at a practice, DSS are placed at a critical and unique juncture to offer immediate care. Therefore, the ability to confidently administer management advice or instructions is vital to the prognosis of an avulsed tooth. Statistical analysis showed that greater age (*p* = 0.0021) and years of experience (*p* = 0.0112) significantly influenced confidence levels of managing avulsion. Ninety percent of those very confident in managing avulsion were between the ages of 40 and 50 and above 50 (30% and 60% respectively). Likewise, eight participants (16.6%) with greater than 10 years of experience reported being very confident in managing avulsion. It is positive that greater life experience and years of exposure to the field allowed participants to compensate for lack of previous avulsion education. However, it is to be noted that 31.7% of participants with over 10 years of experience still reported confidence in managing avulsion to some degree only. Therefore, although all those who felt very confident had over 10 years of experience, for some, years of experience still did not compensate for lack of avulsion education.

Other studies on the impact of education on retention of avulsion knowledge in non-dental cohorts (nursing, speech therapy and dental students, parents, teachers, nurses and physicians) further affirmed high rates of retention [28,29,30,31,32,33]. Therefore, avulsion training and education for all critical groups and especially DSS is successful in helping participants retain knowledge regarding avulsion management. Thus, avulsion training in DSS training programs could significantly enhance participants’ confidence levels in emergency situations. Statistical analysis also revealed that participants’ willingness to receive additional avulsion training was related to receiving educational posters/brochures about avulsion in the past (*p* < 0.0001). International studies assessing the effectiveness of educational posters distributed to students, parents and teachers have demonstrated successful outcomes [28,35,36,37]. Therefore, visual representations of avulsion management appealed to participants as an effective method of education. Furthermore, DSS who had previously encountered posters were more inclined to receive them again, which indicates its positive effect on readers.

Calculating the true sample size of DSS in the Greater Cairns Region and in Australia is another limitation of this study. Data indicate that 50 out of 60 respondents (83.33%) currently have the position of dental assistant. The majority of dental assistants have certificate III and IV (66.66%) and some of them have no dental education (16.67%). While a large proportion of DSS in Greater Cairns Regions and Australia are dental assistants, they are not registered with any government organization or association. The rate of response in this study is 34.1% (60 participants) which can be improved in future studies.

Although data collection was initially intended to be done within controlled, timed and invigilated groups, due to the Covid-19 pandemic, this was not possible. The only viable alternative was online survey. Online survey was performed, although this had a detrimental effect on the response rate, which can be improved significantly.

Despite these limitations, the results of this study provide invaluable knowledge regarding the knowledge base of DSS regarding the dental emergency of avulsion. It is recommended to the Australian Dental Association/Dental Council to provide further training/workshop, educational brochure or CPD course for dental assistants and other health professionals in dental avulsion management. Furthermore, registration of dental assistants with the dental board can assist in controlling and enhancing the quality of their job. Investigation of the knowledge and capability of DSS in management of avulsion injury with a larger number of participants is recommended for future studies.

## 6. Conclusions

This study found that DSS in the Greater Cairns region have a fair grasp surrounding first-aid knowledge of dental avulsion injuries. However, their knowledge was derived from years of work experience rather than avulsion training. This lack of formal education left most DSS feeling under confident in managing an emergency avulsion situation. To improve DSS’ confidence and proficiency in responding to avulsion, formal training and induction programs of DSS in tertiary institutions and public and private practices need to include comprehensive avulsion training. Furthermore, the distribution of informative posters/leaflets to these organizations will also help reinforce appropriate initial management and response to dental avulsion.

## Figures and Tables

**Table 1:** Participants’ demographic profile.

	Number	Percentage (%)
**Gender**		
Male	7	11.7
Female	53	88.3
**Age**		
Below 20	0	0
20 to 39	33	55
40 to 50	12	20
Above 50	15	25
**Years of Experience**		
1–5 years	12	20
5–10 years	18	30
10–15 years	9	15
15–20 year	3	5
More than 20 years	18	30
**Education/Qualification**		
Certificate III in Dental Assisting	29	48.3
Certificate IV in Dental Assisting	11	18.3
Bachelor of Oral Health	7	11.7
Overseas Dental Degree	3	5
No Dental Education/Qualification	6	10
Other	4	6.7
**Current Employment**		
Part time	11	18.3
Full time	30	50
Casual	21	35

**Table 2:** Participants’ training experience.

	Number	Percentage (%)
**Previous Avulsion Training**		
Yes	25	41.7
No	35	58.3
**Received Poster/Brochure about Avulsion Management**		
Yes	13	21.7
No	47	78.3

**Table 3:** Knowledge of initial management to tooth avulsion.

	Number	Percentage (%)
**Urgency for Dental Treatment**		
Immediately	55	91.7
Within few hours when dentist is free	5	8.3
The next day appointment	0	0
Not sure	0	0
**Awareness of Patient’s Dental Age**		
Permanent tooth	49	81.7
Baby tooth	4	6.7
Not sure	7	11.7
**Replantation of Permanent Tooth**		
Yes	55	91.7
No	1	1.7
Not sure	4	6.7
**Replantation of Deciduous Tooth**		
Yes	2	3.3
No	51	85
Not sure	7	11.7
**Management of an Avulsed Tooth Which Appears Soiled**		
Rinse the tooth under running water	6	10
Rinse the tooth with fresh milk	33	55
Wash it with patient saliva outside the mouth	18	30
Gently scrub with toothbrush or a clean tissue	1	1.7
Not sure	2	3.3
**Handling of an Avulsed Tooth While Cleaning**		
Hold from crown	53	88.3
Hold from root	0	0
Doesn’t matter where to hold	2	3.3
Not sure	5	8.3

**Table 4:** Knowledge of subsequent management to tooth avulsion.

	Number	Percentage (%)
**Technique to Transport Tooth**		
Ice pack	1	1.7
Patient’s mouth between cheek and teeth	21	35
Paper tissue	0	0
Liquid medium	33	55
Not sure	5	8.3
**Liquid Medium Used to Transport Tooth**		
Fresh milk	39	65
Fresh water	1	1.7
Patient’s saliva	17	28.3
Saline	2	3.3
Alcohol	0	0
Mouthwash	0	0
Not sure	1	1.7

**Table 5:** Participants’ confidence level towards avulsion management and willingness to receive additional training.

	Number	Percentage (%)
**Confidence Level**		
Very confident	10	16.7
To some degree	40	66.7
Not confident at all	10	16.7
**Willingness to Receive Additional Training**		
Yes	52	86.7
No	8	13.3

**Figure A1:**
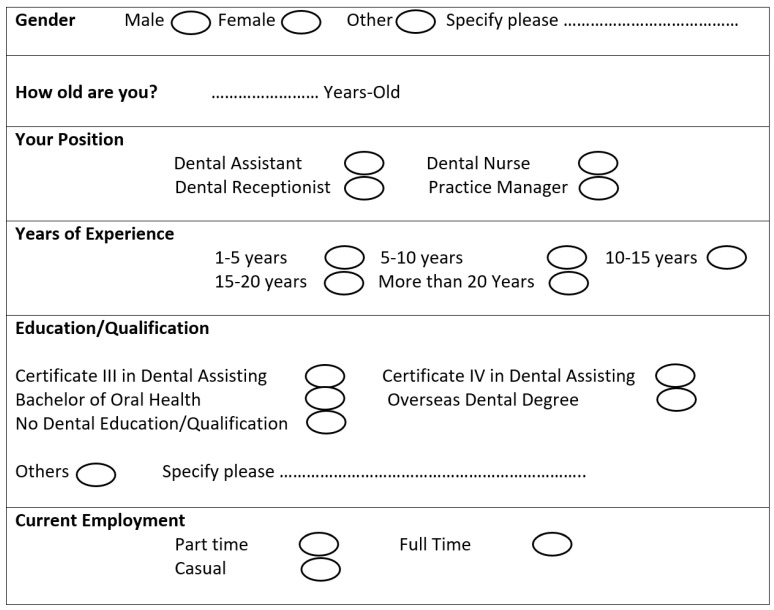
Background survey questionnaire.

## Appendix A. Survey

### Appendix A.1. Demographic Information

Please tick/cross or provide the most appropriate answer.

### Appendix A.2. Knowledge and Management of Avulsed Tooth (Knocked-out Tooth from Socket)

In a normal working day, mother of 9 years-old child calls your clinic and reports her child just had a traumatic accident and one of the front upper teeth is knocked out.

1. How urgently does a child with a knocked-out tooth need dental treatment?
  - Immediately
  - Within few hours when dentist is free
  - The next day appointment
  - Not sure
2. Do you expect the knocked-out tooth in this child is a permanent or baby tooth?
  - Permanent tooth
  - Baby tooth
  - Not sure
3. Should a permanent knocked-out tooth re-implanted into socket?
  - Yes
  - No
  - Not sure
4. Should a baby knocked-out tooth re-implanted into socket?
  - Yes
  - No
  - Not sure
5. If the knocked-out tooth is dirty, what advice do you give to clean the dirt?
  - Rinse the tooth under running water
  - Rinse the tooth with dairy milk
  - Wash it with patient’s saliva outside the mouth
  - Gently scrub with toothbrush or a clean tissue
  - Not sure
6. How should they hold the knocked-out tooth while cleaning?
  - Hold from crown
  - Hold from root
  - Doesn’t matter where to hold
  - Not sure
7. If the knocked-out tooth is not re-implanted into socket, what is the best way to transport it to the clinic?
  - Place it in an ice pack
  - Place it in patient’s mouth between cheek and teeth
  - Wrap it in a paper tissue
  - Place it in a liquid medium (next question)
  - Not sure
8. Which liquid medium is best for storage of the tooth?
  - Fresh dairy milk
  - Fresh water
  - Patient’s saliva
  - Salt solution
  - Alcohol
  - Mouthwash
  - Not sure

### Appendix A.3. Dental Avulsion Education

1. Have you ever had a training or information session for dental avulsion?
  - Yes
  - No
2. Have you ever been supplied with a poster or brochure about management of dental avulsion?
  - Yes
  - No
3. How confident are you in management of dental avulsion?
  - Very confident
  - To some degree
  - Not confident at all
4. Do you feel you need more awareness in management of dental avulsion?
  - Yes
  - No

Thank you for your time.
